# Antecedents of civil engineering students' entrepreneurial intentions: Dataset article

**DOI:** 10.1016/j.dib.2023.109410

**Published:** 2023-07-13

**Authors:** Hicham Lotfi, Khadija Douayri, Houda Bouarir, Omar Boubker

**Affiliations:** a Laayoune Higher School of Technology, Ibn Zohr University, Agadir, Morocco; b Laboratory of Metrology and Information Processing, Ibn Zohr University, Agadir, Morocco; c Higher School of Technology, Mohammed First University, Oujda, Morocco; d Faculty of Law Economic and Social Sciences, Sidi Mohamed Ben Abdellah University, Fez, Morocco

**Keywords:** Students, Civil engineering, Need for achievement, Entrepreneurial intention

## Abstract

The Kingdom of Morocco has implemented significant reform projects over the past decade to promote youth entrepreneurship. In light of these efforts, it is essential to examine the antecedents of entrepreneurial intentions among students. To this end, the current data article aims to explore the antecedents of civil engineering students' entrepreneurial intentions. Thus, we collected data from civil engineering students using a self-administered questionnaire through Google Forms. We employed the partial least squares structural equation modeling (SEM) to analyze the collected dataset. The data analysis using SmartPLS software showed that entrepreneurial attitude, entrepreneurial capacity, and subjective norms positively and significantly affect the civil engineering students' entrepreneurial intention. The insights from this study can be used by Moroccan higher education institutions (HEI) managers to identify crucial elements that can improve students' inclination towards entrepreneurship.

## Nomenclature

AVE: Average variance extracted.
CMV: Common method variance.
f^2^: Effect size.
GoF: Goodness-of-fit index.
HEI: Higher education institutions.
HTMT: Heterotrait-monotrait ratio of correlations
PLS-SEM: Partial least squares structural equation modeling.
Q^2^: Predictive relevance.
R^2^: Coefficient of determination of endogenous constructs
α.: Cronbach's alpha

**Specifications Table**

**Table:** 

Subject	Business, Management and decision sciences
Specific subject area	Entrepreneurship
Type of data	Tables and Figures
How data were acquired	An online survey was carried out among civil engineering students.
Data format	Raw, analyzed and descriptive data
Description of data collection	The link to the survey was distributed through various channels, such as emails and social networks, such as LinkedIn and WhatsApp. Participants were invited to complete the online questionnaire by following the link provided in these messages. This multi-channel distribution strategy allows for greater reach and accessibility for potential respondents.
Data source location	Morocco; Latitude: 31.7945; Longitude: -7.0849.
Data accessibility	Repository name: Zenodo (https://zenodo.org) Data identification number: 10.5281/zenodo.8048415 Direct URL to data: https://doi.org/10.5281/zenodo.8048415

### Value of the Data

• The dataset provides valuable insights into the factors that influence civil engineering students' entrepreneurial intentions in Morocco.
• The dataset can be used to explore the factors that affect the entrepreneurial intention of public university students in other fields of study, such as chemistry or biology, using the measurement scale adopted in the current data article.
• The dataset provides valuable implications for policymakers and educators to design interventions and programs that can enhance civil engineering students' entrepreneurial intentions.
• Researchers who are interested in exploring the factors that influence civil engineering students' entrepreneurial intentions can use this data as a foundation.
• The dataset can serve as a benchmark for future research on entrepreneurial intentions among students in other countries, providing a comparative perspective on the factors that drive entrepreneurial intentions.

### 1 Objective

The dataset is generated to contribute to the ongoing efforts in Morocco to strengthen youth entrepreneurship, by identifying the factors that can influence the entrepreneurial intentions of civil engineering students. The data was gathered using a self-administered questionnaire among civil engineering students in Morocco, and used the partial least squares technique SEM for data analysis. By identifying these critical factors, the study can provide valuable insights for managers in HEI's who are responsible for promoting entrepreneurship among students. They can use the dataset to develop targeted interventions and programs that can enhance students' entrepreneurial intentions and support the country's broader efforts to foster economic growth and job creation through entrepreneurship.Overall, this data article offers insight into antecedents of student entrepreneurial intention and provides actionable implications for policymakers, educators, and professionals interested in fostering civil engineering entrepreneurial intention in Morocco.

### 2 Data Description

**Fig. 1:**
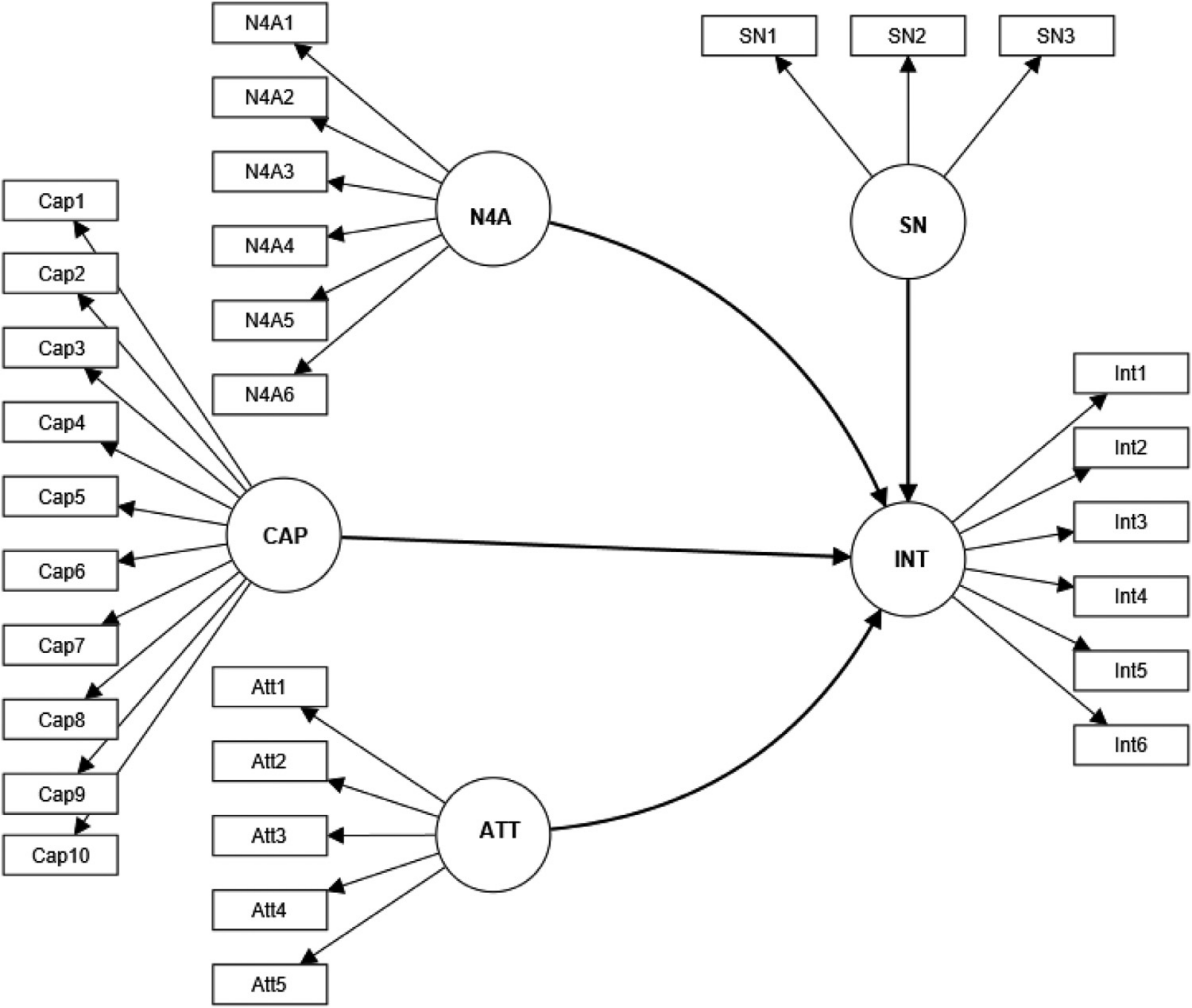
Research model including measurement instruments.

The research model was built based on previous studies [1], [2], [3], [4]. For operationalization of the constructs, we used questions selected from existing literature. Specifically, Need for achievement (N4A) was assessed using a set of 6 items. The perceived entrepreneurial capacity (CAP) was assessed using a set of 10 items [5]. Five items were chosen to measure attitude towards entrepreneurship (ATE) [6]. We selected three items to measure perceived subjective norms (SN) [5]. Students' entrepreneurial intention (INT) was measured using six items [7] (Fig. 1). As well, a 5-item Likert-type scale ranging from one (totally disagree) to five (totally agree) was employed.

Our study population consisted of Moroccan civil engineering students. In order to reach all these students, we used different channels of data collection, such as directly addressing the questionnaire to the concerned students, as well as by contacting the heads of the civil engineering programs in Morocco. At this level, we opted for a probability sampling method, specifically we used the simple random sampling. By using this technique, we ensured that every Moroccan civil engineering student had an equal opportunity to be selected for our study.

Based on ministerial notes published by Morocco's Ministry of Higher Education, Scientific Research and Innovation, the total enrollment of civil engineering students in higher education institutions is estimated at 640 students in 2022-2023. For calculating the sample size, the most commonly employed technique is Krejcie and Morgan's procedure [8]. Thus, based on Krejcie and Morgan's table, we considered a representative sample of 242 civil engineering students for this study.

For data collection, we used a self-administered questionnaire which was first distributed to civil engineering students using the Google Forms tool over a two-month period between February 14 and April 15, 2023. The questionnaire was designed to obtain information on various aspects related to entrepreneurship and the factors that influence students' intentions to become entrepreneurs. During this first phase of data collection, we collected a total of 127 valid responses.

To further increase the sample size, we conducted a second round of data collection between May 31 and June 14, 2023. During this stage, the questionnaire link was emailed to the heads of civil engineering programs in Morocco. During this second phase, we gathered 118 valid responses. Over the two data collection rounds, we gathered a total of 245 eligible responses, which is considered as appropriate and representative sample size.

The collected data was stored in Microsoft Excel spreadsheet (.xlsx), in a secure and organized manner, which facilitated the subsequent analysis of the data using SmartPLS 4 software.

**Fig. 2:**
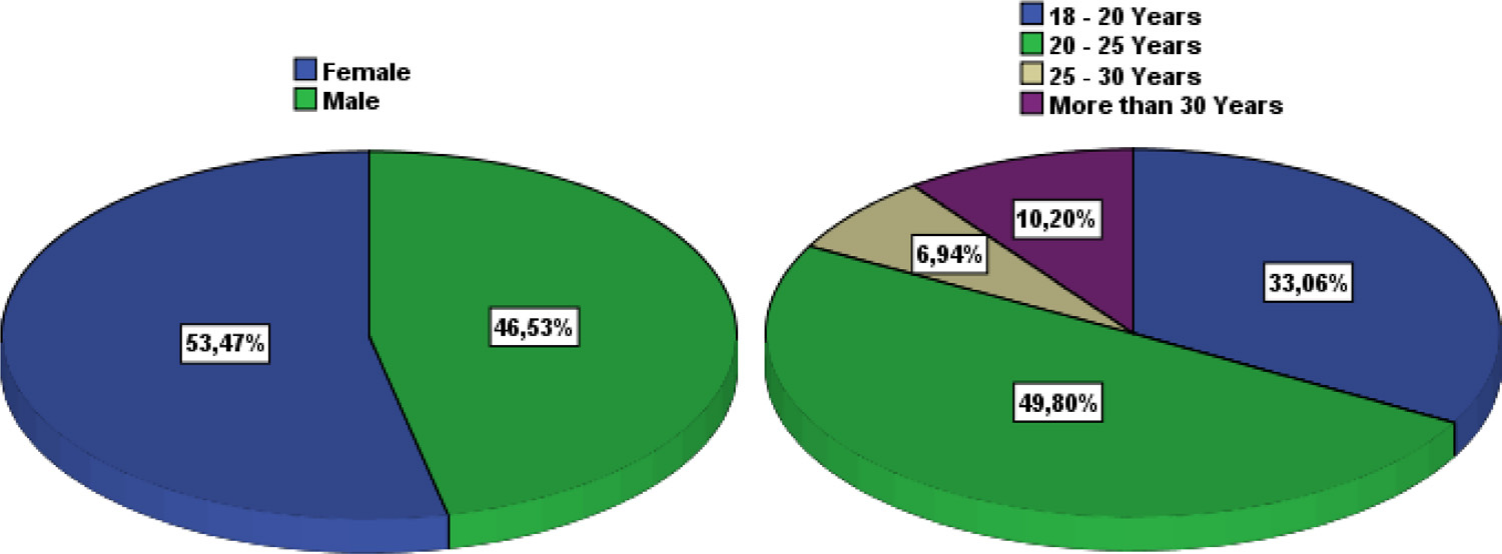
Respondents' gender and age ranges.

**Fig. 3:**
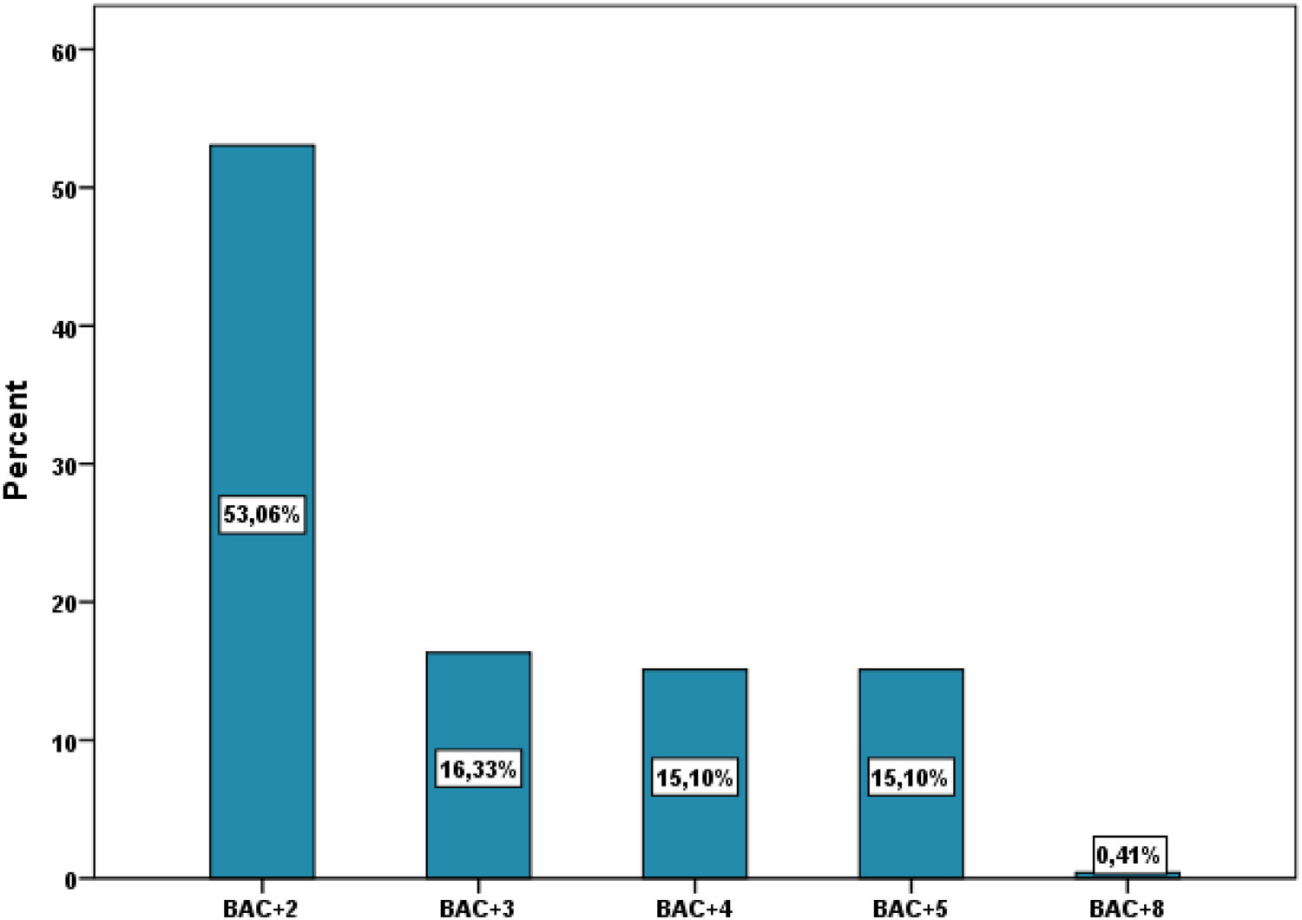
Respondents' educational level.

**Fig. 4:**
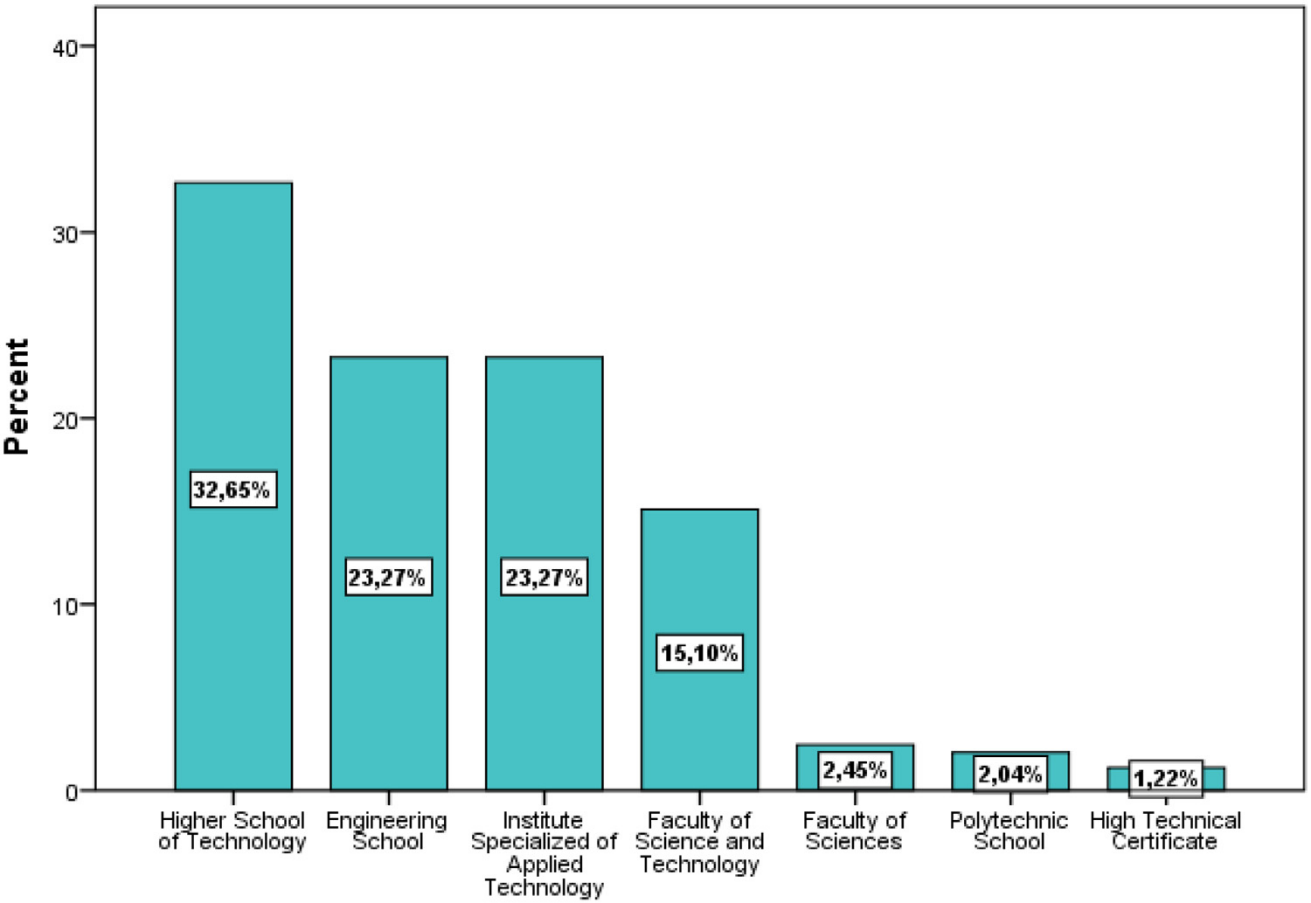
Respondents' institutions.

Table 1 illustrates the socio-demographic characteristics of the survey participants. The variables include gender, age, education level, institution, study field, associative activity, and participation in entrepreneurship competitions. As shown in Fig. 2, more than one-half of the respondents were female (53.47%) and most were between 20 and 25 years old (49.80%). The majority of respondents had a BAC+2 level of education (53.06%), followed by BAC+3 and BAC+4. The highest level of education was BAC+8, which was obtained by only one respondent (Fig. 3). The majority of respondents (94.29%) were from higher school of technology (32.65%), engineering school (23.27%), institute specialized of applied technology (23.27%), and faculty of science and technology (15.10%) (Fig. 4).

**Table 1:** Socio-demographic characteristics of the surveyed students (N=**245**).

Variable	Category	N	%
Gender	Female	131	53.47
	Male	114	46.53

Age	18 - 20 Years	81	33.06
	20 - 25 Years	122	49.80
	25 - 30 Years	17	6.94
	More than 30 Years	25	10.20

Education level	BAC+2	130	53.06
	BAC+3	40	16.33
	BAC+4	37	15.10
	BAC+5	37	15.10
	BAC+8	1	0.41

Institution	Higher School of Technology	80	32.65
	Engineering School	57	23.27
	Institute Specialized of Applied Technology	57	23.27
	Faculty of Science and Technology	37	15.10
	Faculty of Sciences	6	2.45
	Polytechnic School	5	2.04
	High Technical Certificate	3	1.22
Study field	Civil Engineering	220	89.80
	Building	10	4.08
	Topography	9	3.67
	Hydraulic and Environment	6	2.45

Associative activity	Yes	160	65.31
	No	85	34.69

Entrepreneurship competition	No	195	79.59
	Yes	50	20.41

Name of Competition	None	195	79.59
	ISTA Entrepreneurial Innovation Program	30	12.24
	Smart Bank by BMCE of AFRICA	17	6.94
	INJAZ Al-Maghrib	3	1.22

Entrepreneurship and project management Education	Yes	124	50.61
	No	121	49.39

**Fig. 5:**
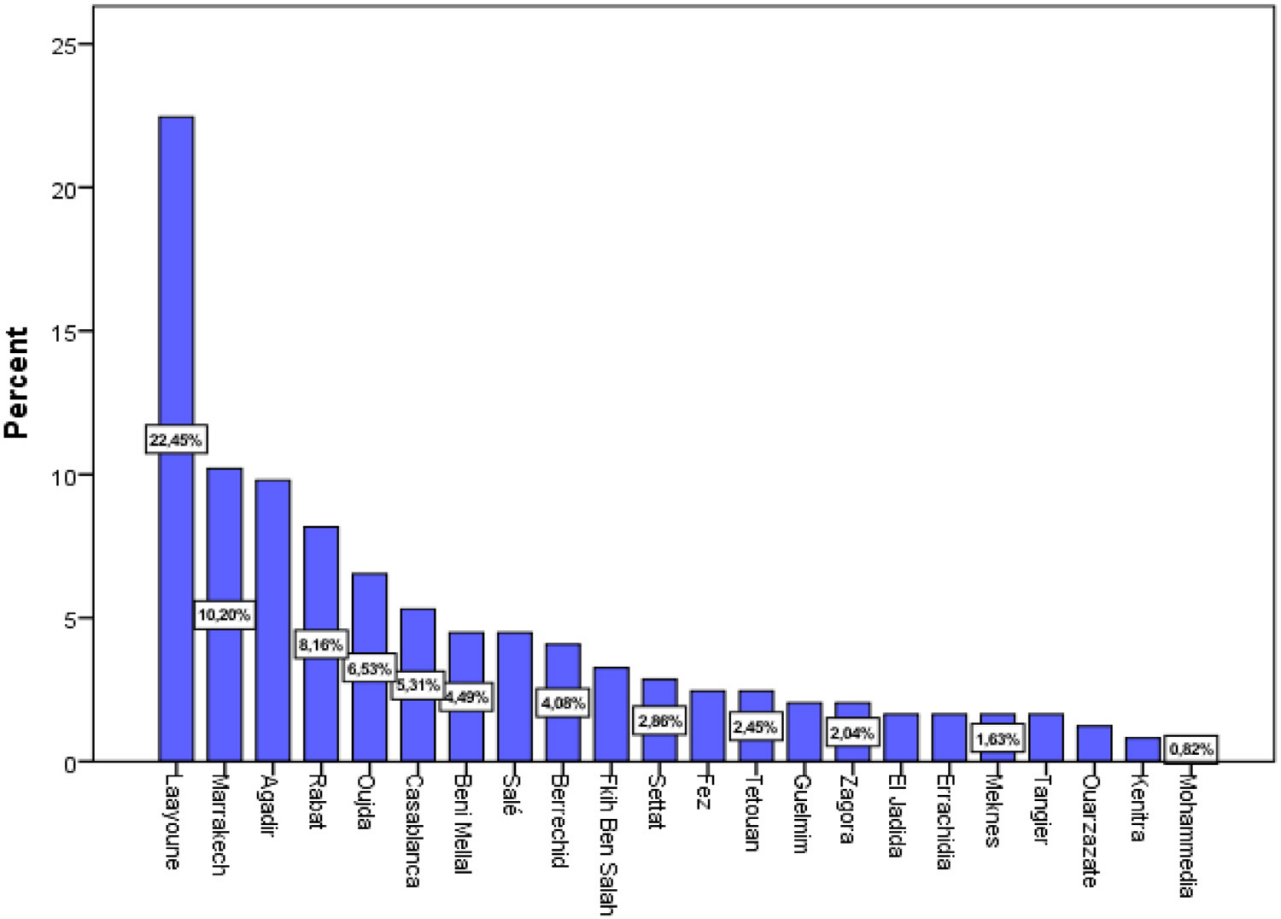
Respondents' cities.

**Fig. 6:**
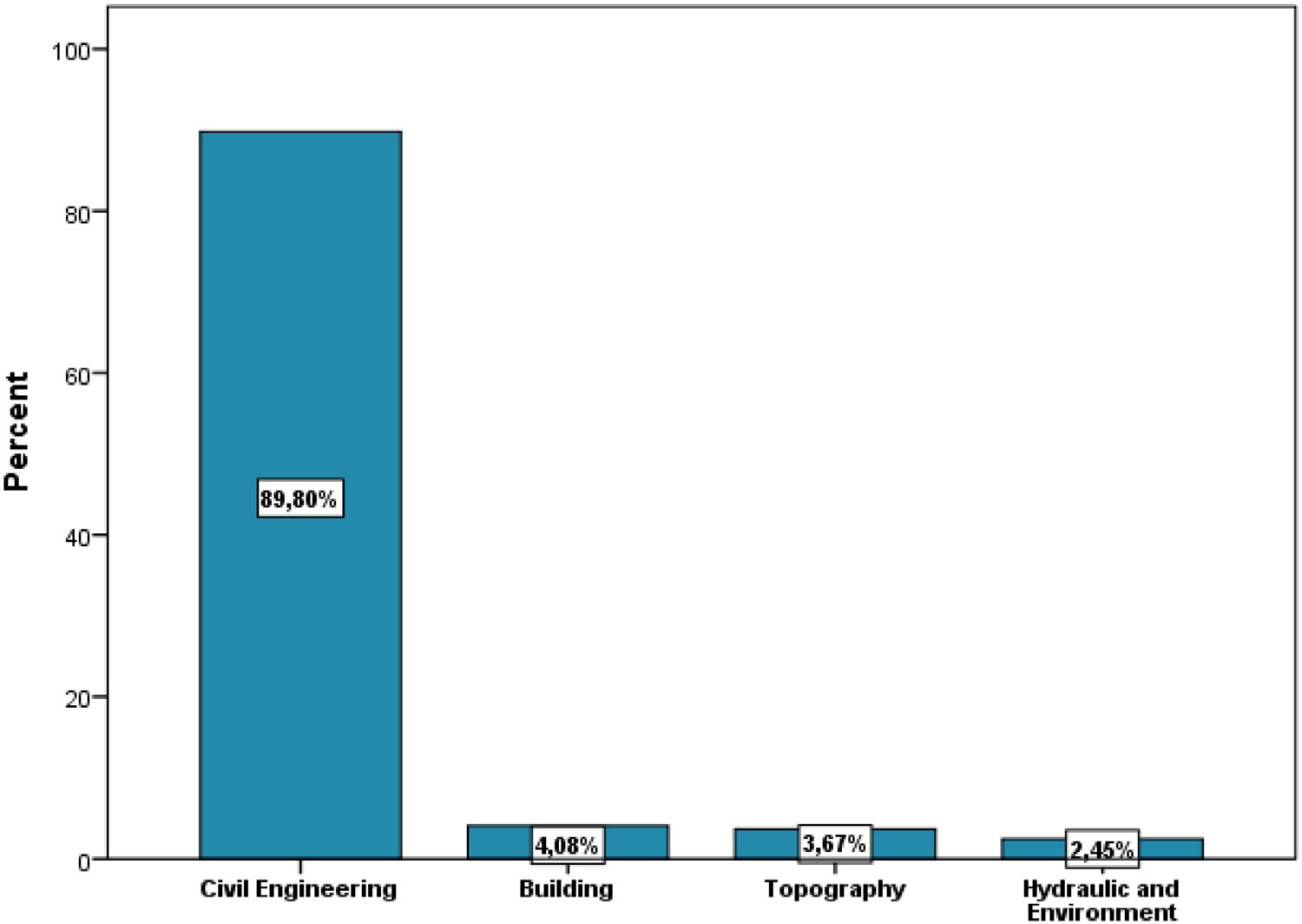
Respondents' field of study.

**Fig. 7:**
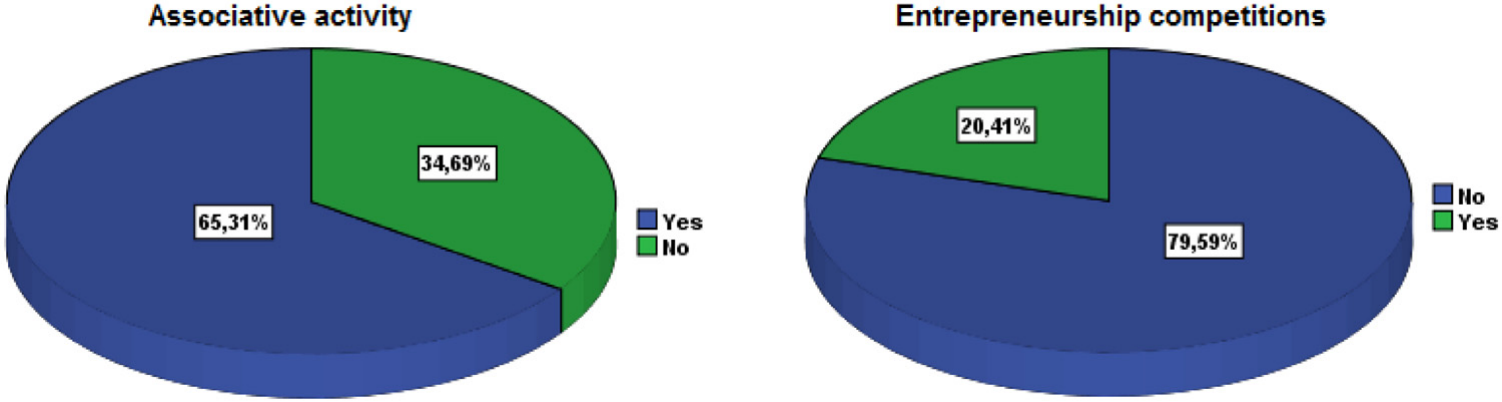
Respondents' involvement in associative activities and entrepreneurship competitions

**Fig. 8:**
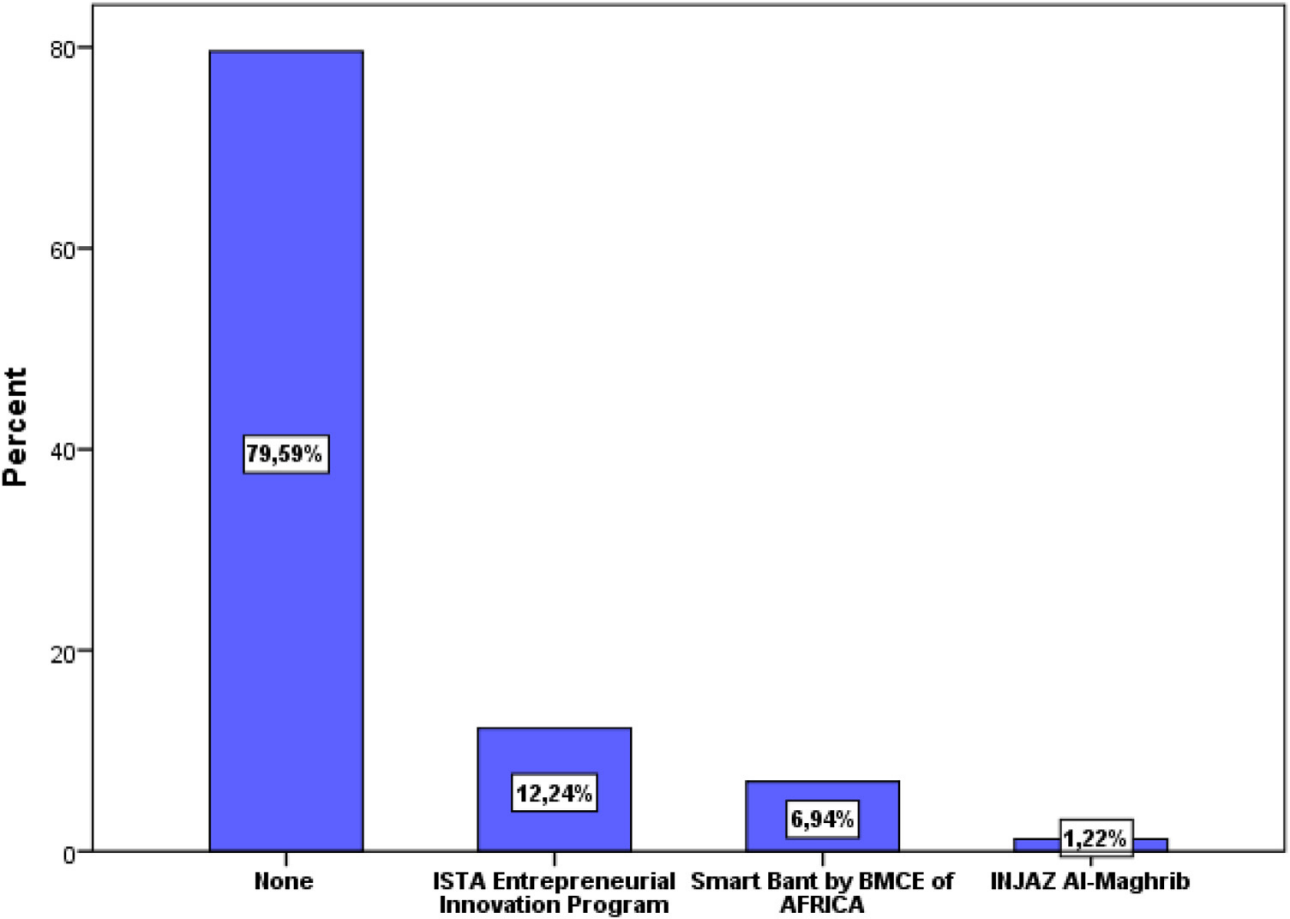
Entrepreneurial competitions attended by study participants

**Fig. 9:**
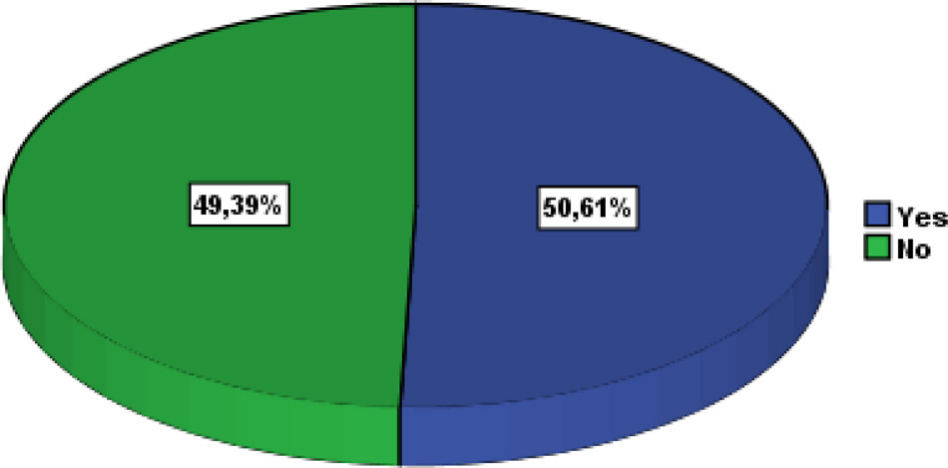
Respondents' classification regarding entrepreneurship and project management education

A large part of the responses (62.44%) were from the cities Laayoune (22.4%), Marrakech (10.2%), Agadir (9.8%), Rabat (8.2%), Oujda (6.5%), and Casablanca (5.3%) (Fig. 5). Civil engineering was the most popular study field (89.80%), followed by building, hydraulic and environment, and topography (Fig. 6). The majority of respondents were engaged in associative activities (65.31%), and 20.41 percent had taken part in entrepreneurship competitions (Fig. 7). The ISTA Entrepreneurial Innovation Program was the competition in which 12.24% of students participated (Fig. 8). More than half (50.61%) of the respondents indicated that they have received education in entrepreneurship and project management (Fig. 9).

### 3 Experimental Design, Materials and Methods

We employed a quantitative research design to test the hypotheses, utilizing the PLS-SEM method with SmartPLS 4 software. Prior to performing data analysis according to PLS-SEM approach, we applied Harman's single-factor test using SPSS to check for the CMV. The outputs of this test indicate that a single factor extracts 46.5% of the total variance, showing the absence of CMV in the collected data.

**Fig. 10:**
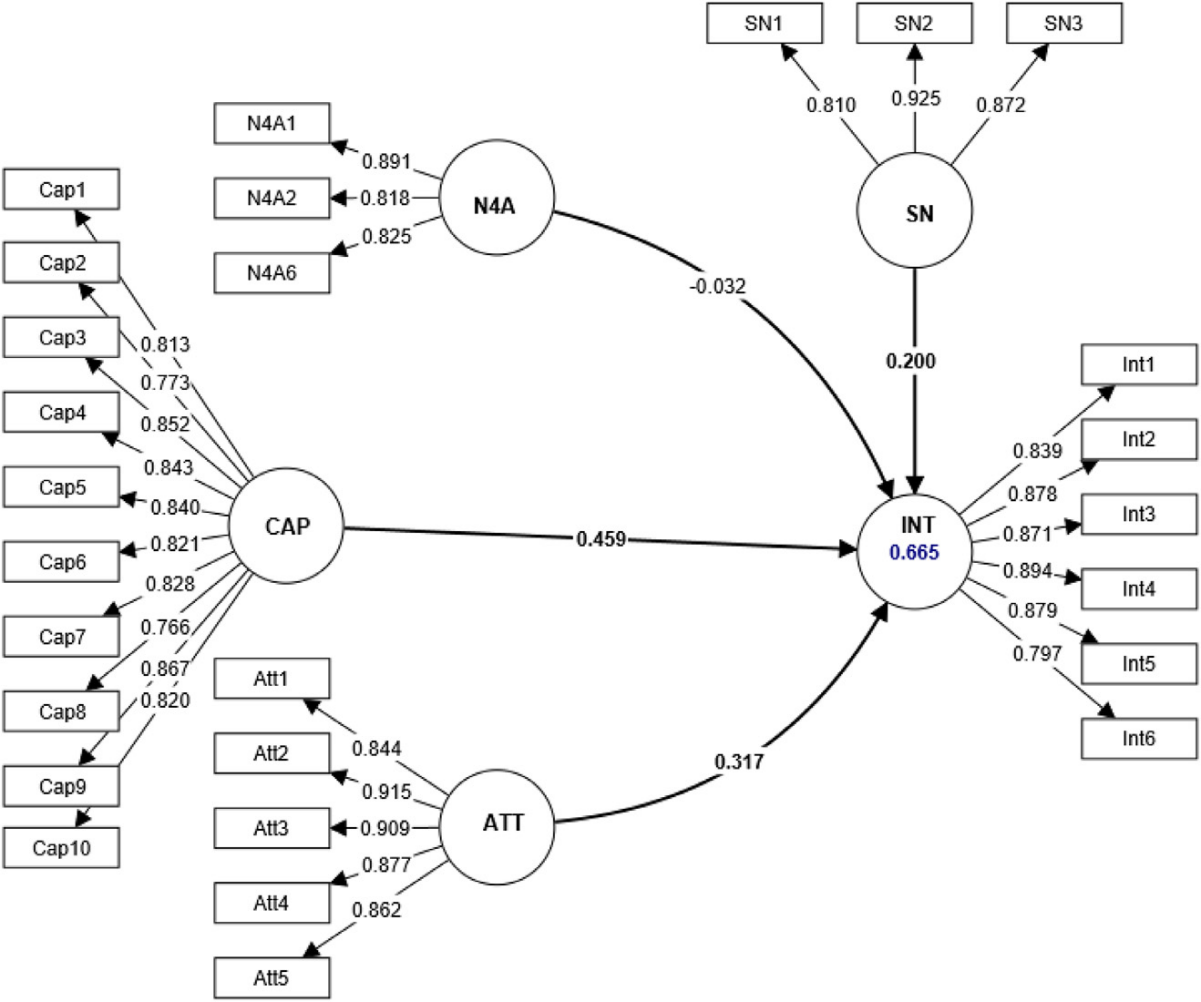
Outer model evaluation.

Applying the PLS-SEM technique requires evaluating both the outer and inner model validity [9]. After removing all items with a low external loading value, i.e., N4A3 (0.313), N4A4 (0.086), and N4A5 (0.212), all remaining item loadings become higher than 0.7 (Fig. 10). Table 2 shows the reflective constructs reliability and convergent validity. The constructs measured in the study are entrepreneurial attitude, entrepreneurial capacity, entrepreneurial intention, need for achievement, and subjective norms. The table includes information about the items used to measure each construct, their outer loadings, Cronbach's alpha (α), the average variance extracted (AVE), and other measures of composite reliability (rho_a, rho_c). The outer loadings range from 0.766 to 0.925, indicating that all items have a strong relationship with their respective construct. Cronbach's alpha values range from 0.800 to 0.947, indicating high internal consistency for each construct. The AVE values range from 0.677 to 0.778, indicating that each construct shares a significant proportion of variance with its measures. The values of the composite reliability criteria including rho_a, and rho_c, are above 0.7, indicating that each construct has a high composite reliability. Hence, these outcomes prove that the measurement constructs have good reliability and convergent validity.

**Table 2:** Reliability and convergent validity assessment.

Constructs	Items	Outer loadings	α	rho_a	rho_c	AVE
Entrepreneurial Attitude	Att1	0.844	0.928	0.937	0.946	0.778
	Att2	0.915				
	Att3	0.909				
	Att4	0.877				
	Att5	0.862				

Entrepreneurial capacity	Cap1	0.813	0.947	0.949	0.954	0.677
	Cap2	0.773				
	Cap3	0.852				
	Cap4	0.843				
	Cap5	0.840				
	Cap6	0.821				
	Cap7	0.828				
	Cap8	0.766				
	Cap9	0.867				
	Cap10	0.820				

Need for achievement	N4A1	0.891	0.800	0.808	0.882	0.714
	N4A2	0.818				
	N4A6	0.825				

Subjective Norms	SN1	0.810	0.838	0.841	0.903	0.757
	SN2	0.925				
	SN3	0.872				

Entrepreneurial Intention	Int1	0.839	0.929	0.931	0.945	0.740
	Int2	0.878				
	Int3	0.871				
	Int4	0.894				
	Int5	0.879				
	Int6	0.797				

Table 3 presents the discriminant validity assessment using two methods: the Fornell-Larcker criterion and the Heterotrait-Monotrait (HTMT) ratio of correlations. The diagonal values of the Fornell-Larcker criterion represent the square root of the average variance extracted (AVE) of each construct, while the off-diagonal values represent the correlations between constructs. According to the Fornell-Larcker criterion, discriminant validity is supported when the square root of the AVE of a construct is greater than its correlation with other constructs. Based on this criterion, the table shows that each construct's square root of AVE is greater than its correlations with other constructs, indicating that the constructs have adequate discriminant validity. The HTMT matrix shows the ratio of correlations between constructs to the correlations within constructs. The HTMT values less than 0.85 indicate that the constructs have good discriminant validity. According to the HTMT matrix, the values between each pair of constructs are less than 0.90, indicating that the constructs have good discriminant validity.

**Table 3:** Discriminant validity assessment using Fornell-Larcker and HTMT matrix.

	Fornell-Larcker criterion					HTMT matrix				
	ATT	CAP	INT	N4A	SN	ATT	CAP	INT	N4A	SN
ATT	**0.882**									
CAP	0.577	**0.823**				0.607				
INT	0.698	0.729	**0.860**			0.744	0.772			
N4A	0.628	0.723	0.635	**0.845**		0.723	**0.816**	0.730		
SN	0.684	0.555	0.649	0.684	**0.870**	0.772	0.613	0.734	0.834	

Table 4 shows the discriminant validity assessment using cross loading. The table displays the cross-loading values for each item on each construct. The diagonal values represent the square root of the AVE for each construct, which is higher than the cross-loading values for the corresponding items.

**Table 4:** Discriminant validity assessment using cross loading.

	ATT	CAP	INT	N4A	SN
Att1	**0.844**	0.476	0.574	0.539	0.630
Att2	**0.915**	0.525	0.730	0.570	0.674
Att3	**0.909**	0.543	0.606	0.558	0.573
Att4	**0.877**	0.496	0.574	0.548	0.577
Att5	**0.862**	0.500	0.568	0.557	0.547
Cap1	0.604	**0.813**	0.669	0.694	0.668
Cap2	0.524	**0.867**	0.658	0.623	0.460
Cap3	0.531	**0.773**	0.625	0.644	0.587
Cap4	0.531	**0.852**	0.653	0.635	0.504
Cap5	0.465	**0.843**	0.624	0.496	0.412
Cap6	0.525	**0.840**	0.571	0.666	0.482
Cap7	0.410	**0.821**	0.550	0.573	0.363
Cap8	0.346	**0.828**	0.558	0.515	0.337
Cap9	0.355	**0.766**	0.490	0.489	0.299
Cap10	0.396	**0.820**	0.559	0.583	0.383
Int1	0.590	0.660	**0.839**	0.548	0.499
Int2	0.610	0.663	**0.878**	0.556	0.593
Int3	0.629	0.581	**0.871**	0.540	0.584
Int4	0.603	0.626	**0.894**	0.552	0.587
Int5	0.645	0.636	**0.879**	0.588	0.615
Int6	0.517	0.595	**0.797**	0.490	0.464
N4A1	0.564	0.650	0.563	**0.891**	0.618
N4A2	0.452	0.482	0.462	**0.818**	0.566
N4A6	0.563	0.679	0.573	**0.825**	0.549
SN1	0.591	0.540	0.551	0.599	**0.810**
SN2	0.624	0.505	0.592	0.650	**0.925**
SN3	0.568	0.403	0.551	0.532	**0.872**

**Fig. 11:**
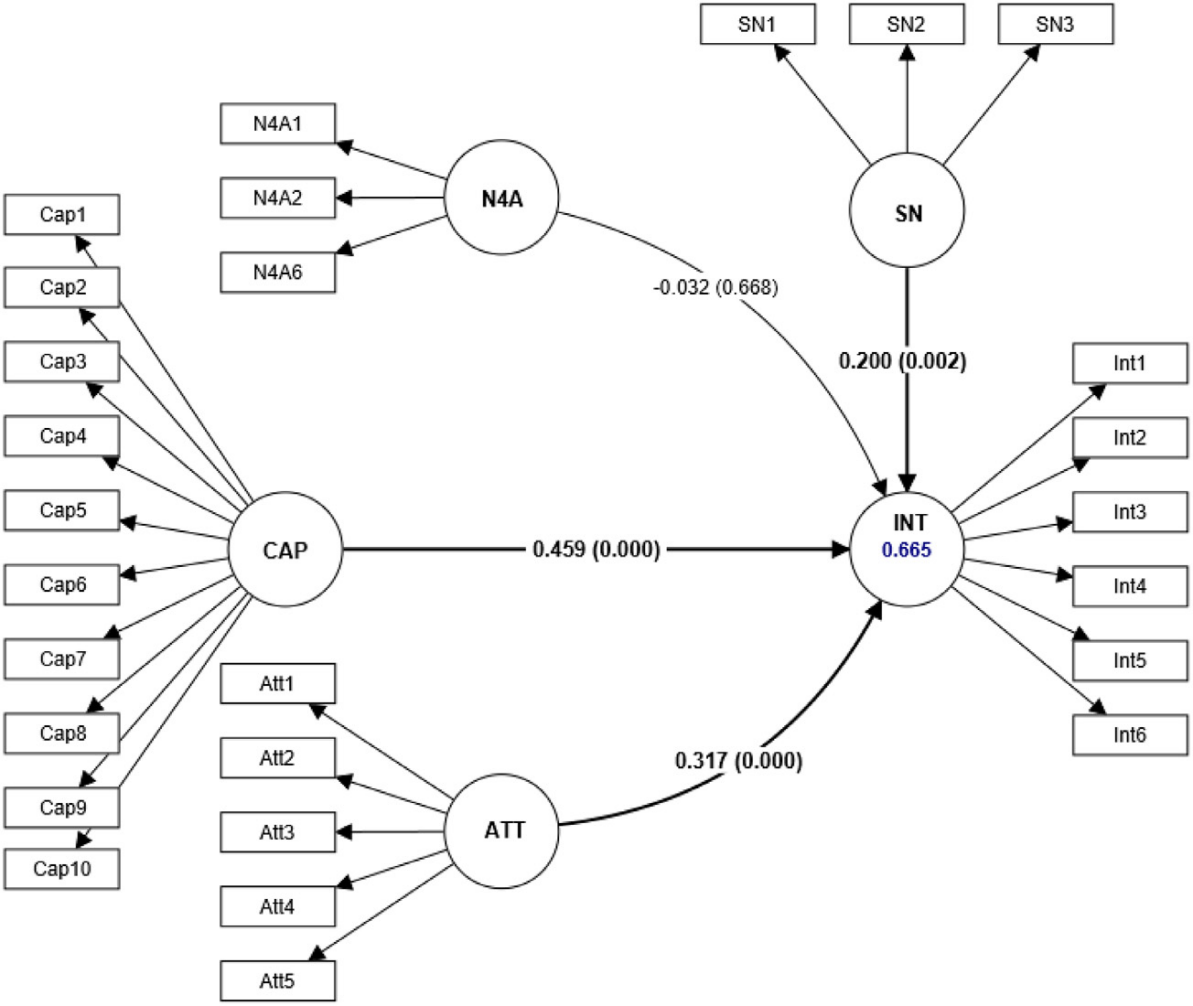
Inner model assessment.

Table 5 shows the outputs of the inner model evaluation for a structural model analyzed using the partial least squares SEM technique. This table presents four hypotheses (H1, H2, H3, and H4) and their respective β values, T statistics, and P values. Based on the SmartPLS 4 outputs H1, H2, and H4 hypotheses are accepted as their P values are less than 0.05, indicating that the relationships between entrepreneurial attitude (β value = 0.317; P value = 0.000), entrepreneurial capacity (β value = 0.459; P value = 0.000), subjective norms (β value = 0.200; P value = 0.002), and entrepreneurial intentions (INT) are statistically significant. These hypotheses also have large f^2^ values, indicating a strong effect size. The R^2^ values of entrepreneurial intentions (0.665) is also relatively high, indicating that the predictor variables explain a significant proportion of the variance in the criterion variable. The Q² (0.646) and GoF (0.698) values are also high, indicating a good predictive relevance and overall fit of the model. On the other hand, H3 is rejected, as its P value (0.668) is greater than 0.05, indicating that the relationship between need for achievement (N4A) and civil engineering entrepreneurial intentions (INT) is not statistically significant (Fig. 11).

**Table 5:** Inner model evaluation.

Hypothesis				β value	T statistics	P values	f^2^	R^2^	Q^2^	GoF	Decision
H1	ATT	→	INT	0.317	4.867	0.000	0.139	0.665	0.646	0.698	Accepted
H2	CAP	→	INT	0.459	7.042	0.000	0.284				Accepted
H3	N4A	→	INT	-0.032	0.429	0.668	0.001				Rejected
H4	SN	→	INT	0.200	3.089	0.002	0.051				Accepted

### Ethics Statement

Participation in this study was voluntary and anonymous. The survey does not include any identifying information about the participants. Everyone who responded to the survey provided his or her informed consent. Ethical approval was not necessary and therefore not sought.

### CRediT authorship contribution statement

**Hicham Lotfi:** Conceptualization, Data curation, Writing – original draft, Visualization, Investigation. **Khadija Douayri:** Writing – review & editing, Data curation. **Houda Bouarir:** Writing – review & editing, Visualization, Investigation. **Omar Boubker:** Supervision, Conceptualization, Methodology, Formal analysis, Software, Writing – review & editing.

## Declaration of Competing Interest

The authors declare that they have no known competing financial interests or personal relationships, which have or could be perceived to have influenced the work reported in this article.

## Data Availability

Dataset on civil engineering students' entrepreneurial intentions (Original data) (Zenodo).
